# The Relationship Between Body Mass Index and In-Hospital Mortality in Patients Following Coronary Artery Bypass Grafting Surgery

**DOI:** 10.3389/fcvm.2021.754934

**Published:** 2021-10-08

**Authors:** Gabby Elbaz-Greener, Guy Rozen, Shemy Carasso, Fabio Kusniec, Merav Yarkoni, Ibrahim Marai, Bradley Strauss, Harindra C. Wijeysundera, Frank W. Smart, Eldad Erez, Ronny Alcalai, David Planer, Offer Amir

**Affiliations:** ^1^Department of Cardiology, Hadassah Medical Center, Jerusalem, Israel; ^2^Faculty of Medicine, Hebrew University of Jerusalem, Jerusalem, Israel; ^3^Cardiology Division, Hillel Yaffe Medical Center, Hadera, Israel; ^4^The Ruth and Bruce Rappaport Faculty of Medicine, Technion, Haifa, Israel; ^5^Cardiology Division, Harvard Medical School, Massachusetts General Hospital, Boston, MA, United States; ^6^Division of Cardiovascular Medicine, Baruch Padeh Medical Center, Poriya, Israel; ^7^The Azrieli Faculty of Medicine in the Galilee, Bar-Ilan University, Safed, Israel; ^8^Division of Cardiology, Schulich Heart Centre, Sunnybrook Health Sciences Centre, University of Toronto, Toronto, ON, Canada; ^9^Louisiana State University School of Medicine, New Orleans, LA, United States; ^10^Department of Cardio Surgery, Hadassah Medical Center, Hebrew University of Jerusalem, Jerusalem, Israel

**Keywords:** body mass index (BMI), coronary artery bypass grafting surgery (CABG), ischemic heart, outcome, mortality

## Abstract

**Background:** The association between Body Mass Index (BMI) and clinical outcomes following coronary artery bypass grafting (CABG) remains controversial. Our objective was to investigate the real-world relationship between BMI and in-hospital clinical course and mortality, in patients who underwent CABG.

**Methods:** A sampled cohort of patients who underwent CABG between October 2015 and December 2016 was identified in the National Inpatient Sample (NIS) database. Outcomes of interest included in-hospital mortality, peri-procedural complications and length of stay. Patients were divided into 6 BMI (kg/m^2^) subgroups; (1) under-weight ≤19, (2) normal-weight 20–25, (3) over-weight 26–30, (4) obese I 31–35, (5) obese II 36–39, and (6) extremely obese ≥40. Multivariable logistic regression model was used to identify predictors of in-hospital mortality. Linear regression model was used to identify predictors of length of stay (LOS).

**Results:** An estimated total of 48,710 hospitalizations for CABG across the U.S. were analyzed. The crude data showed a U-shaped relationship between BMI and study population outcomes with higher mortality and longer LOS in patients with BMI ≤ 19 kg/m^2^ and in patients with BMI ≥40 kg/m^2^ compared to patients with BMI 20–39 kg/m^2^. In the multivariable regression model, BMI subgroups of ≤19 kg/m^2^ and ≥40 kg/m^2^ were found to be independent predictors of mortality.

**Conclusions:** A complex, U-shaped relationship between BMI and mortality was documented, confirming the “obesity paradox” in the real-world setting, in patients hospitalized for CABG.

## Introduction

Body mass index (BMI) is widely used for routine characterization of weight status in epidemiological and clinical research, however with some limitations, for instance not distinguishing fat from muscle mass (1). The World Health Organization (WHO) classifies a patient body weight by six categorical subgroups, (1) under-weight, BMI ≤ 18.5 kg/m^2^; (2) normal-weight, BMI 18.5–24.9 kg/m^2^; (3) over-weight, BMI 25–29.9 kg/m^2^; (4) obese class I, BMI 30–34.9 kg/m^2^; (5) obese class II, BMI 35–39.9 kg/m^2^; and (6) extremely obese BMI ≥ 40 Kg/m^2^ (1, 2). In population-based studies, higher BMI was associated with increased incidence of major cardiovascular risk factors (1, 3). BMI has been proven an independent risk factor for various cardiovascular conditions such as acute coronary syndrome, congestive heart failure, sudden cardiac death, atrial and ventricular arrhythmia and stroke (3, 4).

Several studies investigated the effect of different BMI arrays on short- and long-term mortality rates post cardiac surgery with contradictory conclusions. Some studies suggested that obesity was an independent negative predictor of morbidity and mortality (5–8) while others showed more favorable outcomes after cardiac surgery in obese patients (9–13). This phenomenon has been referred to as the obesity paradox (9–13). One explanation for this paradox is that patients with higher BMI may have a lower postoperative bleeding volume and transfusion rate; thus, such patients may save blood products during on-pump CABG factor that contributes to higher survival rates (14). On the other hand, sternal wound infections are more prevalent in obese patients and prior studies have shown increased morbidity and mortality rates in obese patients (15–17).

In the current study, we aimed to describe the BMI distribution and in-hospital outcomes among different BMI subgroups post-CABG, during the index hospitalization.

## Methods

### Data Source

The data were drawn from the National Inpatient Sample (NIS), the Healthcare Cost and Utilization Project (HCUP), and Agency for Healthcare Research and Quality (AHRQ) (18, 19). The NIS database only includes de-identified data; therefore, this study was considered exempt from institutional review by the Human Research Committee.

The NIS is the largest collection of data on all-payer hospitalizations in the United States (U.S.), and represents an approximate 20% stratified sample of all inpatient discharges from U.S. hospitals (20). This includes information at the hospital-level, such as hospital region, teaching status, bed size and cost of hospitalization, and other data at the patient-level, including demographic characteristics, primary and secondary diagnoses and procedures, comorbidities and length of stay (LOS). National estimates can be calculated using the patient-level and hospital-level sampling weights that are provided by the HCUP.

For the purpose of this study, we obtained data for the years 2015 and 2016. The International Classification of Diseases, 10th Revision, Clinical Modification (ICD-10-CM) was used from the last quarter of 2015 and thereafter for reporting diagnoses and procedures in the NIS database during the study period. For each index hospitalization, the database provides a principal discharge diagnosis and a maximum of 14 or 24 additional diagnoses, in addition to a maximum of 15 procedures. We restricted our cohort to the period during which data was coded with ICD-10 codes, because the ICD-10 system includes individual codes for BMI values and ranges.

### Study Population and Variables

We identified patients 18 years of age or older with a primary diagnosis of CABG procedure based on ICD-10-CM code starting with I10.PR1 with 0210-0213xxx codes, who have one of the Z68.x codes as I10-Dx1 to I10-Dx30. These codes represent the six subgroups in our study; Z68, Z68.20–25, Z68.26–30, Z68.31–35, Z68.36–39, and Z68.4 (BMI ≤ 19 under-weight group; BMI 20–25 normal-weight group; BMI 26–30 over-weight group; BMI 31–35 obese I group, BMI 36–39 obese II group and BMI equal or ≥40 extremely obese group, respectively).

The following patient demographics were collected from the database: age, sex, and race. Prior comorbidities were identified by measures from the AHRQ. For the purpose of calculating Deyo-Charlson Comorbidity Index (Deyo-CCI), additional comorbidities were identified from the database using ICD-10-CM codes. Deyo-CCI is a modification of the Charlson Comorbidity Index, containing 17 comorbid conditions of differential weights, with a total score ranging from 0 to 33 (detailed information on Deyo-CCI is provided in the Appendix Table 1). Higher Deyo-CCI scores indicate a greater burden of comorbid diseases and is associated with mortality 1-year after admission (21). The index has been used extensively in studies from administrative databases, with proved validity in predicting short- and long-term outcomes (22, 23).

Our primary outcome in this study was in-hospital mortality. The secondary outcomes included in-hospital periprocedural complications and length of stay in the hospital. In-hospital complications were defined as previously reported (24–26) and included known cardiac surgery related complications as follows: (1) Pericardial complications as tamponade, hemopericardium, pericarditis and pericardiocentesis; (2) Cardiac complications (during or resulting from procedure), defined as cardiac block, myocardial infarction, cardiac arrest, congestive heart failure, cardiogenic shock and others; (3) Pulmonary complications, defined as pneumothorax/hemothorax, diaphragm paralysis, post-operative respiratory failure, and other iatrogenic respiratory complications; (4) Vascular complications, defined as accidental puncture or laceration during a procedure, injury to blood vessels, arteriovenous fistula, injury to retroperitoneum, vascular complication requiring surgical repair, re-open, and other vascular complications; (5) Infection, defined as fever, septicemia, and post-procedural aspiration pneumonia; (6) Neurological, defined as nervous system complication, unspecified, central nervous system complication, iatrogenic cerebrovascular infarction or hemorrhage cerebrovascular effect, and transient ischemic attack; (7) Acute renal failure; (8) Cardiogenic shock; (9) Diaphragmatic paralysis; (10) Re-open surgery; (11) Permanent pacemaker implantation (PPM); (12) New onset atrial fibrillation; (13) Wound infection.

Detailed information on all ICD-10-CM codes used to identify in-hospital complications, is summarized in the Appendix Table 2.

### Statistical Analysis

The chi-square (χ^2^) and Wilcoxon Rank Sum tests were used to compare categorical variables and continuous variables, respectively. The NIS provides discharge sample weights that are calculated within each sampling level as the ratio of discharges in the universe to discharges in the sample (25). We generated a weighted logistic regression model to identify independent predictors of in-hospital mortality. Candidate variables included patient-level characteristics, Deyo-CCI and hospital-level factors. We included all candidate variables that were associated with our primary and secondary outcome in our final multivariable regression model. A linear regression model was used to identify predictors of LOS.

For all analyses, we used SAS^®^ software version 9.4 (SAS Institute Inc., Cary, NC.) A *p* < 0.05 was considered statistically significant.

## Results

### Study Cohort

A total sample of 9,742 CABG hospitalizations across the U.S. during 2015 (last quarter) and 2016 were included in the analysis. After implementing the weighting method, these represented an estimated total of 48,710 hospitalizations for CABG, in patients who had BMI information during the index hospitalization. The majority of patients (70.1%) were male, and the mean age of the cohort was 63.4 ± 22.1 years. A smaller proportion of CABG patients were underweight with a BMI ≤ 19 kg/m^2^ (Table 1).

**Table 1:** Frequency distribution of baseline characteristics by BMI group in CABG patients.

**BMI groups (Kg/m^2^)**	**≤19**	**20–25**	**26–30**	**31–35**	**36–39**	**≥40**	**Total**	***P*-value**
	**Under-weight**	**Normal-weight**	**Over-weight**	**Obese I group**	**Obese II group**	**Extremely obese**		
Unweighted^a^	137	263	1,570	3,544	1,952	2,276	9,742	
Weighted^b^	685	1,315	7,850	17,720	9,760	11,380	48,710	
Age group, %, years								<0.001
18-44	1.5	0.8	1.8	3.0	4.8	5.2	3.6	
45-59	20.4	22.8	24.7	29.7	31.2	34.0	29.9	
60-74	50.4	48.7	54.5	53.8	54.0	53.1	53.6	
≥ 75	27.7	27.8	19.0	13.5	10.0	7.6	12.9	
Gender, %								<0.001
Male	67.2	71.1	72.9	74.6	68.6	62.5	70.1	
Female	32.8	28.9	27.1	25.3	31.3	37.5	29.9	
Race, %								<0.001
White	70.1	71.9	73.6	76.6	75.4	78.1	76.0	
Non-white	25.5	17.9	19.7	15.9	16.3	15.4	16.7	
Comorbidity, %								
Hypertension	48.9	58.6	66.2	67.3	67.9	65.4	66.3	<0.001
Congestive heart failure	21.9	15.2	13.9	11.8	13.8	17.2	14.1	<0.001
Diabetes mellitus	16.8	34.2	36.9	41.4	44.9	50.1	42.9	<0.001
Chronic renal disease	28.5	24.7	20.8	20.3	21.8	24.3	21.9	<0.001
Chronic obstructive pulmonary disease	47.4	23.6	22.7	21.9	24.4	27.0	24.1	<0.001
Peripheral vascular disease	30.7	20.5	17.8	14.3	13.1	12.0	14.5	<0.001
Atrial Fibrillation/Flutter	40.9	31.9	33.0	31.8	33.5	35.6	33.3	<0.001
Prior MI	20.4	22.1	19.3	19.7	18.9	18.9	19.3	0.085
Deyo-CCI, %								<0.001
1	9.5	18.3	20.0	19.9	20.6	17.6	19.3	
2 or higher	87.6	74.5	70.3	69.2	70.7	75.8	71.6	

*MI, Myocardial Infraction; Deyo-CCI, Deyo-Charlson Comorbidity Index*.

a *Represents the number of observations in the NIS database*.

b *Represents total national estimates after applying sampling weights*.

### Patient Characteristics, Presentation of Acute Myocardial Infraction, and Treatment Approach by BMI Group

Study population baseline and clinical characteristics are presented in detail in Tables 1, 2. Male predominance and higher prevalence of comorbidities such as chronic obstructive pulmonary disease, chronic renal disease, atrial fibrillation/flutter and higher Deyo-CCI scores were observed in both under-weight (BMI ≤ 19 kg/m^2)^ and extremely obese groups (BMI ≥ 40 kg/m^2^; Table 1). Forty-five percent of the patients underwent coronary angiogram and CABG in the same index hospitalization. These patients presented more frequently with NSTEMI compared to STEMI. A very small portion (3%) underwent CABG concomitant with valve surgery.

**Table 2:** Frequency distribution of clinical course and outcomes by BMI group.

	**≤19**	**20–25**	**26–30**	**31–35**	**36–39**	**≥40**	**Total**	***P*-value**
	**Under-weight**	**Normal-weight**	**Over-weight**	**Obese I group**	**Obese II group**	**Extremely obese**		
NSTEMI, %	38.7	33.5	29.4	27.9	28.1	29.7	28.9	<0.0001
STEMI, %	10.2	7.2	5.6	5.4	4.6	4.1	5.1	<0.0001
Coronary angiography, %	44.5	47.5	46.9	45.5	42.0	44.4	44.8	<0.0001
Percutaneous coronary intervention, %	1.5	1.9	2.2	2.7	2.2	2.5	2.4	0.048
Concomitant valve surgery, %	3.6	3.8	2.9	2.5	3.1	3	2.9	0.005
Mortality, %	4.4	1.5	1.3	1.0	1.0	2.2	1.4	<0.0001
LOS (days), Mean ± SEM	14.89 ± 0.77	10.54 ± 0.55	9.18 ± 0.16	8.83 ± 0.10	9.00 ± 0.13	10.69 ± 0.18	9.51 ± 0.09	<0.0001

*LOS, Length of Stay; NSTEMI, Non-ST Segment Elevation Myocardial Infraction; STEMI, ST segment Myocardial Infraction*.

### Peri-Procedural Complications

Peri-procedural complications in the study population are reported in Appendix Table 3. A U-shaped relation between the BMI and several periprocedural complications was noted including in acute renal failure, infectious, pulmonary, and vascular complications. In most instances, the underweight population had the highest prevalence of complications including acute renal failure, new atrial fibrillation, cardiac complications, cardiogenic shock, need for a pacemaker, and pulmonary complications (Appendix Table 3).

### Length of Stay and Mortality by BMI Groups

**Figure 1:**
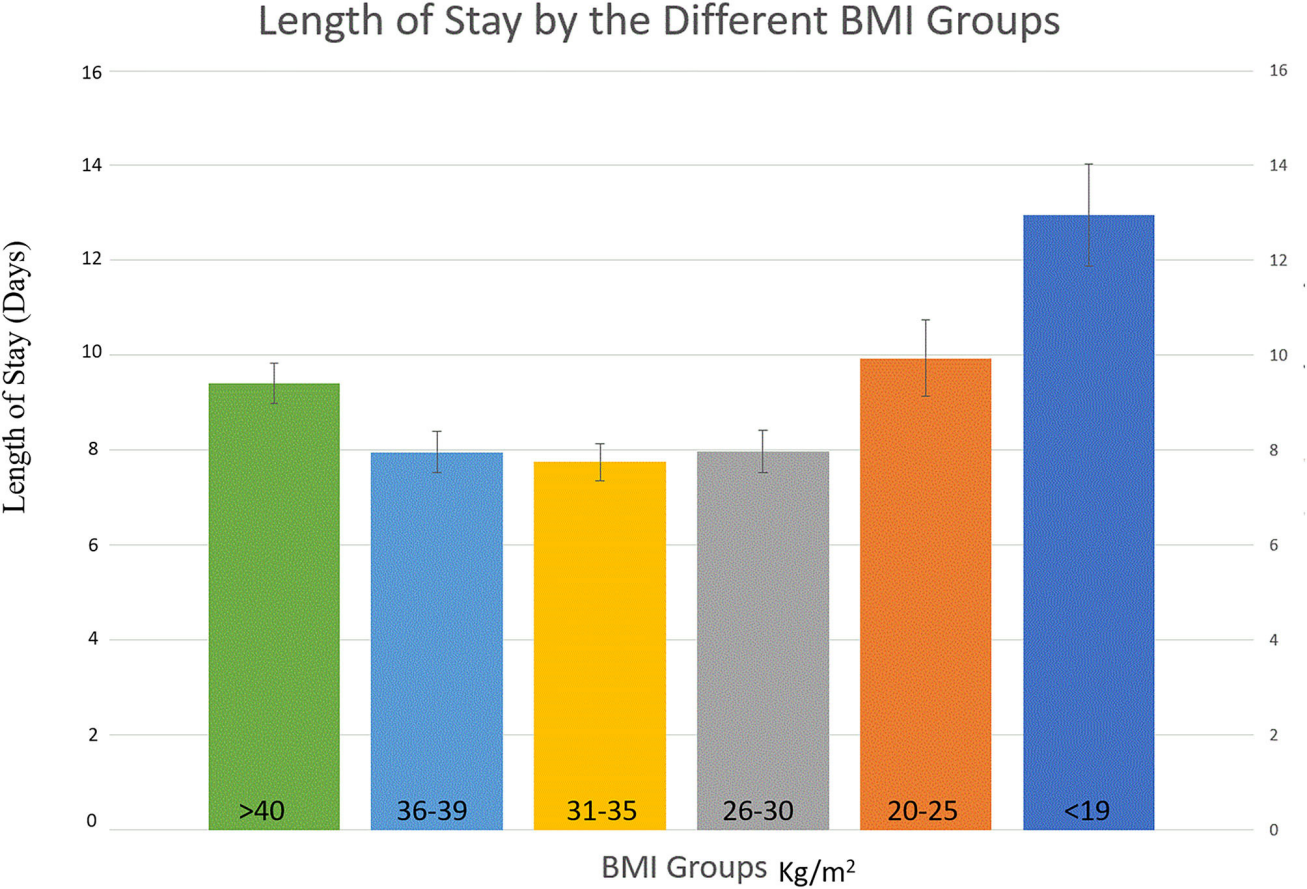
Length of Stay per BMI groups.

**Figure 2:**
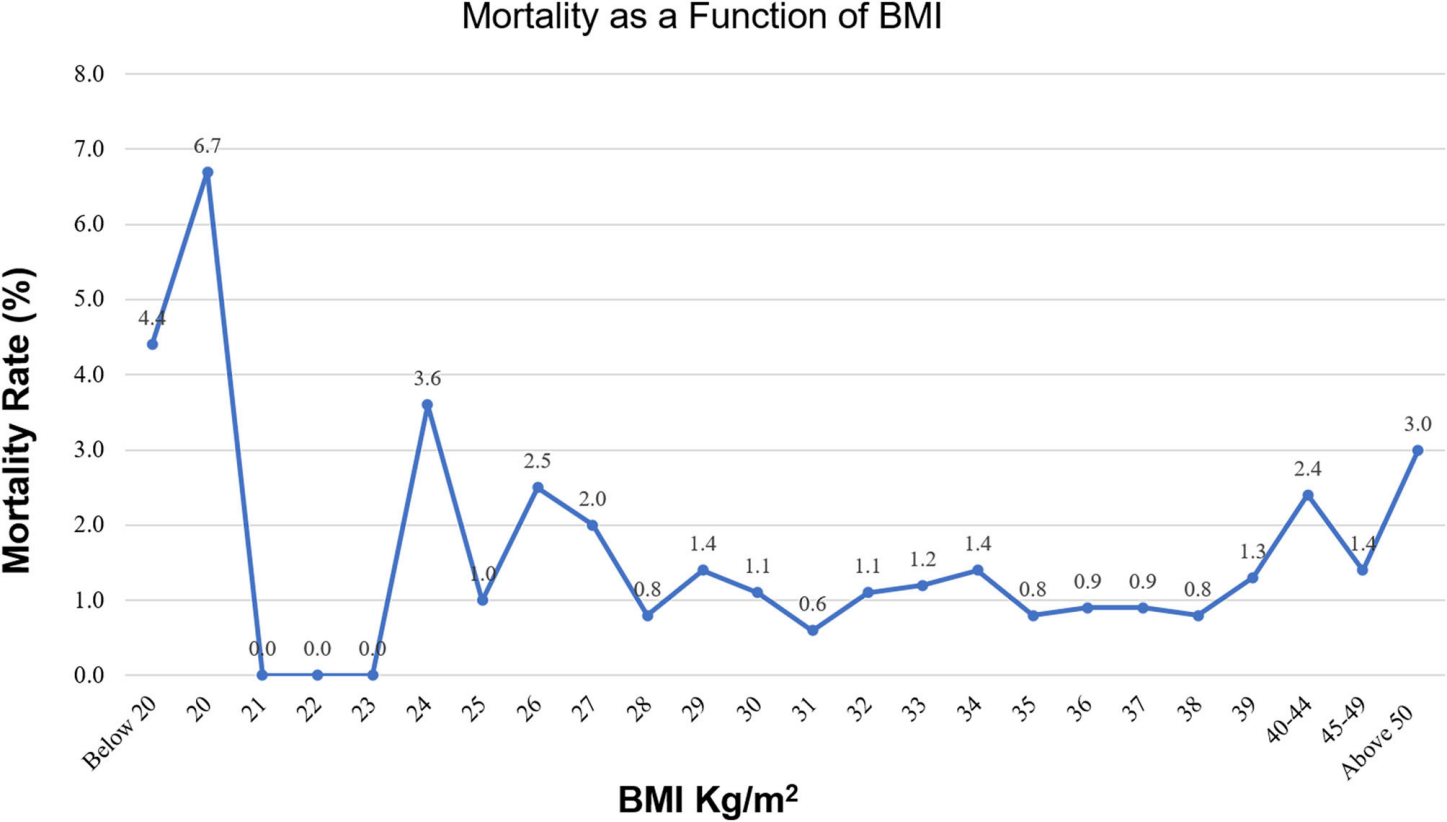
Mortality as a function of BMI.

We observed a complex U-shaped relationship between the BMI and the study outcomes. Both the extremely obese (BMI ≥ 40 kg/m^2^) and underweight patients (BMI ≤ 19 kg/m^2^) had a higher crude mortality and longer LOS (Figure 1). The relationship between mortality and BMI as continuous variable is presented in Figure 2. The overall rate of total mortality during the study period was 1.4% with a significantly higher mortality rate in under-weight (4.4%) and in extremely obese (2.2%) patient population groups (*p* < 0.001; Figure 1). The mean LOS in the total cohort was 9.51 ± 0.09 days. Longer LOS was documented in the under-weight (14.9 ± 0.77 days) and in the extremely obese subgroup (10.7 ± 0.18 days; Figure 2).

### Predictors of In-hospital Mortality

In the univariate analysis, we found that baseline characteristics as older age, increasing Deyo-CCI score, chronic renal failure, atrial fibrillation/flutter, congestive heart failure, peripheral vascular disease, female sex, chronic obstructive pulmonary disease and prior sternotomy all increased the odds of in-hospital mortality (*p* < 0.001; Table 3).

**Table 3:** Univariate analysis for predictors of in-hospital mortality, 2015–2016.

**Predictor**	**Odds ratio** **(95% CI)**	***P*-value**
BMI kg/m^2^ group		<0.001
≤ 19	2.97 (1.67–5.27)	<0.001
20–25	1.00 (reference)	N/A
26–30	0.88 (0.54–1.42)	0.596
31–35	0.66 (0.42–1.06)	0.085
36–39	0.64 (0.39–1.03)	0.068
≥40	1.45 (0.92–2.30)	<0.001
Age group, years		<0.001
18–44	1.00 (reference)	N/A
45–59	1.70 (0.89–3.23)	0.106
60–74	2.41 (1.28–4.53)	0.006
≥75	5.02 (2.65–9.51)	<0.001
Deyo-CCI		<0.001
0	0.43 (0.22–0.84)	0.01
1	1.00 (reference)	N/A
2 or higher	3.39 (2.54–4.52)	<0.001
Gender		<0.001
Male	1.00 (reference)	N/A
Female	1.66 (1.42–1.93)	<0.001
Race		0.42
Non-white	1.00 (reference)	N/A
White	0.92 (0.75–1.13)	0.42
**Comorbidities**		
Atrial fibrillation/flutter		<0.001
No	1.00 (reference)	N/A
Yes	2.08 (1.79–2.42)	<0.001
Chronic obstructive pulmonary disease		0.02
No	1.00 (reference)	N/A
Yes	1.22 (1.03–1.45)	0.02
Congestive heart failure		<0.001
No	1.00 (reference)	N/A
Yes	1.90 (1.59–2.28)	<0.001
Diabetes mellitus		0.009
No	1.00 (reference)	N/A
Yes	1.22 (1.05–1.42)	0.009
Hypertension		<0.001
No	1.00 (reference)	N/A
Yes	0.42 (0.36–0.49)	<0.001
Peripheral vascular disease		<0.001
No	1.00 (reference)	N/A
Yes	2.78 (2.36–3.28)	<0.001
Chronic renal failure		<0.001
No	1.00 (reference)	N/A
Yes	2.54 (2.18–2.97)	<0.001
**Clinical Course**		
NSTEMI		<0.001
No	1.00 (reference)	N/A
Yes	2.09 (1.79–2.43)	<0.001
STEMI		<0.001
No	1.00 (reference)	N/A
Yes	3.12 (2.50–3.88)	<0.001
Percutaneous coronary intervention		<0.001
No	1.00 (reference)	N/A
Yes	2.58 (1.87–3.57)	<0.001
Prior sternotomy		0.021
No	1.00 (reference)	N/A
Yes	1.23 (1.03–1.48)	0.02
Concomitant valve surgery		<0.001
No	1.00 (reference)	N/A
Yes	4.05 (3.14–5.21)	<0.001
VF/VT		<0.001
No	1.00 (reference)	N/A
Yes	9.17 (7.79–10.81)	<0.001

*BMI, Body Mass Index; Deyo-CCI, Deyo-Charlson Comorbidity Index; NSTEMI, Non-ST Segment Elevation Myocardial Infraction; STEMI, ST segment Myocardial Infraction; VF/VT, Ventricular Flutter/Ventricular Fibrillation*.

The U-shaped relationship between BMI and outcomes were again seen in the multivariable analyses. BMI ≤ 19 kg/m^2^ and BMI ≥ 40 kg/m^2^ remained independent predictors of in-hospital mortality after adjusting for potential confounders, with a higher risk of mortality in the underweight patients compared to extremely obese patients (OR 2.64; [95% CI: 1.48–4.71], *p* < 0.001 vs. OR 1.71; [95% CI: 1.07–2.72], *p* = 0.025, respectively; Table 4).

**Table 4:** Multivariable analysis for predictors of in-hospital mortality, 2015–2016.

**Predictor**	**Odds ratio (95% CI)**	***P*-value**
BMI kg/m^2^ group		<0.001
20–25	1.00 (reference)	N/A
≤ 19	2.64 (1.48–4.71)	<0.001
26–30	0.86 (0.52–1.40)	0.541
31–35	0.70 (0.43–1.12)	0.140
36–39	0.76 (0.47–1.25)	0.288
≥40	1.71 (1.07–2.73)	0.025
Age group, years		<0.001
18–44 years	1.00 (reference)	N/A
45–59 years	1.68 (0.88–3.21)	0.116
60–74 years	2.32 (1.23–4.38)	0.009
75 years or older	4.53 (2.37–8.66)	<0.001
Deyo-CCI		<0.001
1	1.00 (reference)	N/A
2 or higher	3.46 (2.50–4.77)	<0.001
Gender		<0.001
Male	1.00 (reference)	N/A
Female	1.42 (1.21–1.68)	<0.001
Race		0.470
Non-white	1.00 (reference)	N/A
White	0.93 (0.75–1.14)	0.470
**Comorbidities**		
Atrial fibrillation/flutter		<0.001
No	1.00 (reference)	N/A
Yes	1.80 (1.53–2.12)	<0.001
COPD		0.208
No	1.00 (reference)	N/A
Yes	0.89 (0.74–1.07)	0.208
Congestive heart failure		0.002
No	1.00 (reference)	N/A
Yes	1.36 (1.13–1.65)	0.002
Diabetes mellitus		0.718
No	1.00 (reference)	N/A
Yes	0.97 (0.82–1.14)	0.718
Hypertension		<0.001
No	1.00 (reference)	N/A
Yes	0.56 (0.47–0.66)	<0.001
Peripheral vascular disease		<0.001
No	1.00 (reference)	N/A
Yes	2.25 (1.89–2.68)	<0.001
Chronic renal failure		<0.001
No	1.00 (reference)	N/A
Yes	1.72 (1.45–2.03)	<0.001
**Clinical course**		
NSTEMI		<0.001
No	1.00 (reference)	N/A
Yes	1.62 (1.38–1.90)	<0.001
STEMI		<0.001
No	1.00 (reference)	N/A
Yes	2.99 (2.36–3.77)	<0.001
Percutaneous coronary intervention		<0.001
No	1.00 (reference)	N/A
Yes	2.22 (1.56–3.16)	<0.001
Prior sternotomy		0.224
No	1.00 (reference)	N/A
Yes	1.13 (0.93–1.36)	0.224
Concomitant valve surgery		<0.001
No	1.00 (reference)	N/A
Yes	3.12 (2.36–4.12)	<0.001
VF/VT		<0.001
No	1.00 (reference)	N/A
Yes	8.02 (6.72–9.57)	<0.001

*BMI, Body Mass Index; Deyo-CCI, Deyo-Charlson Comorbidity Index; NSTEMI, Non-ST Segment Elevation Myocardial Infraction; STEMI, ST segment Myocardial Infraction; VF/VT, Ventricular Flutter/Ventricular Fibrillation*.

### Predictors of LOS

Linear regression models for LOS are represented in the Appendix Tables 4, 5. BMI ≤ 19 kg/m^2^ was found to be an independent predictor of longer hospitalization [OR = 12.95; (95% CI: 11.88–14.03), *p* < 0.001]. Other predictors for longer LOS were older age, Deyo-CCI score ±2, atrial fibrillation, congestive heart failure, and chronic renal failure. Additionally, were patients who presented with acute coronary syndrome and who underwent percutaneous coronary intervention or concomitant valve surgery in the same index hospitalization (Appendix Tables 4, 5).

## Discussion

Utilizing data from the NIS, the largest all-payer inpatient database in the U.S., we identified a weighted total of 48,710 patients to investigate the relationship between BMI and in-hospital outcomes among patients undergoing CABG. To our knowledge, this is the single largest study analyzing the relationship between BMI and CABG in-hospital outcomes. This nationwide data analysis revealed a U-shaped relationship between the BMI and in-hospital mortality during hospitalization for CABG in the U.S. during the study period. BMI ≤ 19 kg/m^2^ and BMI ≥ 40 kg/m^2^ were both independent predictors of higher mortality and longer length of stay in patients post CABG in the U.S. during the study period.

Several studies investigated the effect of different BMI arrays on short- and long-term mortality with contradictory results. Some studies suggested that obesity was an independent negative predictor of morbidity and mortality (5–8) while other showed more favorable outcomes after cardiac surgery in obese patients (9–13). This could partially be explained by the poor association between obese and non-obese groups in comparing morbidity and mortality; many of the studies lacked the “under-weight” and “severe obesity” patient populations in their statistical comparisons of different BMI groups.

Obesity is a rising global epidemic affecting 10–20% of the world's adult population (24), and is further known to complicate anesthesia (27) and surgery with fatal and non-fatal events. Prior studies showed higher rates of surgical site infections in obese patients, venous thromboembolism, more significant blood loss and the need of blood transfusions, as well as longer surgical times (28, 29). Other studies showed increased rates of urine and respiratory infections and post-surgery myocardial infarction events in obese patients (29). Specifically, after CABG surgery, obesity is a strong risk factor for deep sternal wound infections and as such serves an indicator of higher morbidity rates (30). Currently, there is a lack of publications studying cachexia and survival rates in post CABG patients.

On the contrary to the abovementioned studies, others showed that higher weight individuals conferred better survival after surgery. One possible explanation is that high BMI may be beneficial by providing nutritional and caloric reserves in severely and critically ill patients. This is supported by previous studies in additional chronic, debilitating cardiovascular and non-cardiovascular conditions, in which both under-weight and normal weight BMI was associated with increased mortality rates, compared to higher-BMI groups (3, 31–33). Further studies supported that severe cardiovascular disease, such as heart failure, results in tissue hypoperfusion and cardiac cachexia. The hypothesis is that this state resulting from a heightened metabolic or increased catabolic state is associated with worse prognosis (34, 35). On the other hand, elevated BMI might indicate a better metabolic reserve and tolerance to metabolic stress and consequently to a better prognosis (36).

Our study results improve the available literature on the negative association of extremely obese BMI subgroups and early mortality following CABG. Of note, the Society of Thoracic Surgeons (STS) uses statistical models to create risk-adjusted performance metrics for Adult Cardiac Surgery Database (ACSD) participants. BSA and BMI were both independently associated with mortality, and O'Brien et al. (26) found that inclusion of both variables was needed to capture variation in the residuals. In line with our findings, they also demonstrated a U-shape relationship between BMI and mortality (26), reflected in the STS score. In contrast, BMI has not been directly included in the current European (EuroSCORE II) score (37). We believe that this important correlation between BMI and mortality should be reflected in all surgical risk scores.

Contrary to many previous clinical studies, we included the “real world” BMI spectrum including the underweight, normal weight and extremely obese patients for a complete representation. Our results showed that underweight patients undergoing CABG (BMI ≤ 19 kg/m^2^) have the highest risk of mortality compared to the other BMI groups.

This study population represents the entire, nationwide population of patients who underwent CABG in the U.S between October 2015 and December 2016, hence eliminating the “selection bias” in some of the prior studies regarding CABG patients.

The current study should be interpreted in the contexts of several limitations. First, the NIS database is a retrospective administrative database that contains discharge-level records and as such is susceptible to coding errors. The NIS database does not include detailed information about the patients' clinical characteristics, medication, blood tests such as level of cholesterol and markers of inflammation, all of which have been independently linked with adverse cardiovascular events, therefore we cannot rule out residual confounding of the associations we observed. Unfortunately, no data about the number/type of diseased vessels (SYNTAX score etc.), the number of coronary bypass and the techniques preferred (venous vs. arteriosus graft, off-pump BH, etc.) have been included in our study, despite of increasing interest. In addition, the lack of patient identifiers in the NIS precluded us from using other outcome variables and mortality measures such as at 30-days, we could only capture events that occurred in the same index hospitalization. These limitations are counterbalanced by the real world, nationwide nature of the data, as well as mitigation of reporting bias introduced by selective publication of results from specialized centers.

In conclusion, a U-shaped relationship between BMI and mortality was documented in patients hospitalized for CABG in the recent years. Accordingly, BMI should be addressed and considered as part of the risk assessment for in-hospital mortality in patients who are planned for CABG.

## Data Availability Statement

The datasets presented in this study can be found in online repositories. The names of the repository/repositories and accession number(s) can be found in the article/Supplementary Material.

## Ethics Statement

Ethical review and approval was not required for the study on human participants in accordance with the local legislation and institutional requirements. Written informed consent for participation was not required for this study in accordance with the national legislation and the institutional requirements.

## Author Contributions

All authors listed have made a substantial, direct, and intellectual contribution to the work, and approved it for publication.

## Conflict of Interest

The authors declare that the research was conducted in the absence of any commercial or financial relationships that could be construed as a potential conflict of interest.

## Publisher's Note

All claims expressed in this article are solely those of the authors and do not necessarily represent those of their affiliated organizations, or those of the publisher, the editors and the reviewers. Any product that may be evaluated in this article, or claim that may be made by its manufacturer, is not guaranteed or endorsed by the publisher.
