# Supplementary material for: The Relationship Between Body Mass Index and In-Hospital Mortality in Patients Following Coronary Artery Bypass Grafting Surgery
Source: Front Cardiovasc Med. 2021 Oct 8;8:754934. doi: 10.3389/fcvm.2021.754934 (PMC8531483; doi:10.3389/fcvm.2021.754934)
Supplement: Supplementary file 1 [file Data_Sheet_1.docx]

**Appendix**

**Appendix Table 1.** Deyo Comorbidity index

| **ICD-10 CM codes** | **Condition** | **Score** |
| --- | --- | --- |
| I21.x, I22.x, I25.2 | Myocardial infarction | 1 |
| I09.9, I11.0, I13.0, I13.2, I25.5, I42.0, I42.5-I42.9, I43.x, I50.x, P29.0 | Congestive heart failure | 1 |
| I70.x, I71.x, I73.1, I73.8, I73.9, I77.1, I79.0,  I79.2, K55.1, K55.8, K55.9, Z95.8, Z95.9 | Peripheral vascular disease | 1 |
| G45.x, G46.x, H34.0, I60.x-I69.x | Cerebrovascular disease | 1 |
| F00.x-F03.x, F05.1, G30.x, G31.1 | Dementia | 1 |
| I27.8, I27.9, J40.x-J47.x, J60.x-J67.x, J68.4, J70.1, J70.3 | Chronic pulmonary disease | 1 |
| M05.x, M06.x, M31.5, M32.x-M34.x, M35.1, M35.3, M36.0 | Rheumatologic disease | 1 |
| K25.x-K28.x | Peptic ulcer disease | 1 |
| B18.x, K70.0-K70.3, K70.9, K71.3-K71.5, K71.7, K73.x, K74.x, K76.0, K76.2-K76.4, K76.8, K76.9, Z94.4 | Mild liver disease | 1 |
| E10.0, E10.l, E10.6, E10.8, E10.9, E11.0, E11.1, E11.6, E11.8, E11.9, E12.0, E12.1, E12.6, E12.8, E12.9, E13.0, E13.1, E13.6, E13.8, E13.9, E14.0, E14.1, E14.6, E14.8, E14.9 | Diabetes | 1 |
| E10.2-E10.5, E10.7, E11.2-E11.5, E11.7, E12.2-E12.5, E12.7, E13.2-E13.5, E13.7, E14.2-E14.5, E14.7 | Diabetes with chronic complications | 2 |
| G04.1, G11.4, G80.1, G80.2, G81.x, G82.x, G83.0-G83.4, G83.9 | Hemiplegia or paraplegia | 2 |
| I12.0, I13.1, N03.2-N03.7, N05.2-N05.7, N18.x, N19.x, N25.0, Z49.0-Z49.2, Z94.0, Z99.2 | Renal disease | 2 |
| C00.x-C26.x, C30.x-C34.x, C37.x-C41.x, C43.x, C45.x-C58.x, C60.x-C76.x, C81.x-C85.x, C88.x, C90.x-C97.x | Any malignancy including leukemia and lymphoma | 2 |
| I85.0, I85.9, I86.4, I98.2, K70.4, K71.1, K72.1, K72.9, K76.5, K76.6, K76.7 | Moderate or severe liver disease | 3 |
| C77.x-C80.x | Metastatic solid tumor | 6 |
| B20.x-B22.x, B24.x | Acquired Immunodeficiency syndrome (AIDS) | 6 |

**Appendix Table 2.** In-Hospital Complication Codes for ICD-10-CM

| **Complication** | **ICD-10-CM Code(s)** |
| --- | --- |
| **Pericardial** |  |
| Hemopericardium | I31.2 |
| Tamponade | [I31.4](https://www.icd10data.com/ICD10CM/Codes/I00-I99/I30-I52/I31-/I31.4) |
| Pericardiocentesis | OW9DXXX, OW9CXXX, 0W9D40Z |
| Acute pericarditis | I30.1, I30.8, I30.9 |
| **Cardiac**  (including postoperative cardiac block, myocardial infarction,  cardiac arrest, and congestive heart failure) | I97.710, I97.110, I97.120, I97.130, I97.190, I97.88, I97.89, I97.711, I97.790  I97.XXX |
| **Pulmonary** |  |
| Pneumothorax/hemothorax | [J95.811](https://www.icd10data.com/ICD10CM/Codes/J00-J99/J95-J95/J95-/J95.811), [J95.812](https://www.icd10data.com/ICD10CM/Codes/J00-J99/J95-J95/J95-/J95.812), J95.830, J95.831, J94.2 |
| Diaphragm paralysis | [J98.6](https://www.icd10data.com/ICD10CM/Codes/J00-J99/J96-J99/J98-/J98.6) |
| Post-operative Respiratory Failure | [J95.821](https://www.icd10data.com/ICD10CM/Codes/J00-J99/J95-J95/J95-/J95.821), [J96.00](https://www.icd10data.com/ICD10CM/Codes/J00-J99/J96-J99/J96-/J96.00), [J95.822](https://www.icd10data.com/ICD10CM/Codes/J00-J99/J95-J95/J95-/J95.822), [J96.20](https://www.icd10data.com/ICD10CM/Codes/J00-J99/J96-J99/J96-/J96.20) |
| Other iatrogenic Respiratory Complications | J95.88, J95.89, J95.851, J95.859 |
| **Vascular** |  |
| Accidental puncture or laceration during a procedure | [I97.51](https://www.icd10data.com/ICD10CM/Codes/I00-I99/I95-I99/I97-/I97.51), [I97.52](https://www.icd10data.com/ICD10CM/Codes/I00-I99/I95-I99/I97-/I97.52) |
| Injury to blood vessels | S25.X, S35.X |
| Arteriovenous Fistula | I77.0 |
| Injury to retroperitoneum | [S36.899A](https://www.icd10data.com/ICD10CM/Codes/S00-T88/S30-S39/S36-/S36.899A) |
| Vascular complication requiring surgical/Percutaneous repair | [03QY0ZZ](https://www.icd10data.com/ICD10PCS/Codes/0/3/Q/Y/03QY0ZZ), [03QY3ZZ](https://www.icd10data.com/ICD10PCS/Codes/0/3/Q/Y/03QY3ZZ), [03QY4ZZ](https://www.icd10data.com/ICD10PCS/Codes/0/3/Q/Y/03QY4ZZ), [04QY0ZZ](https://www.icd10data.com/ICD10PCS/Codes/0/4/Q/Y/04QY0ZZ), [04QY3ZZ](https://www.icd10data.com/ICD10PCS/Codes/0/4/Q/Y/04QY3ZZ), [04QY4ZZ](https://www.icd10data.com/ICD10PCS/Codes/0/4/Q/Y/04QY4ZZ)  [05QY0ZZ](https://www.icd10data.com/ICD10PCS/Codes/0/5/Q/Y/05QY0ZZ), [05QY3ZZ](https://www.icd10data.com/ICD10PCS/Codes/0/5/Q/Y/05QY3ZZ), [05QY4ZZ](https://www.icd10data.com/ICD10PCS/Codes/0/5/Q/Y/05QY4ZZ), [06QY0ZZ](https://www.icd10data.com/ICD10PCS/Codes/0/6/Q/Y/06QY0ZZ), [06QY3ZZ](https://www.icd10data.com/ICD10PCS/Codes/0/6/Q/Y/06QY3ZZ), [06QY4ZZ](https://www.icd10data.com/ICD10PCS/Codes/0/6/Q/Y/06QY4ZZ),  [02QW0ZZ](https://www.icd10data.com/ICD10PCS/Codes/0/2/Q/W/02QW0ZZ), [02QW3ZZ](https://www.icd10data.com/ICD10PCS/Codes/0/2/Q/W/02QW3ZZ), [02QX4ZZ](https://www.icd10data.com/ICD10PCS/Codes/0/2/Q/X/02QX4ZZ), [03Q00ZZ](https://www.icd10data.com/ICD10PCS/Codes/0/3/Q/0/03Q00ZZ), [03Q03ZZ](https://www.icd10data.com/ICD10PCS/Codes/0/3/Q/0/03Q03ZZ), 03Q04ZZ,  03Q10ZZ, 03Q13ZZ, 03Q14ZZ, 03Q20ZZ, 03Q23ZZ, 03Q24ZZ, 03Q30ZZ, 03Q40ZZ, 03Q33ZZ, 03Q43ZZ, 03Q44ZZ, 03Q50ZZ, 03Q53ZZ, 03Q54ZZ, 03Q60ZZ, 03Q63ZZ, 03Q64ZZ, 03Q74ZZ 03Q70ZZ, 03Q73ZZ, 03Q80ZZ, 03Q83ZZ, 03Q84ZZ, 03Q90ZZ, 03Q93ZZ, 03Q94ZZ, 03QA0ZZ, 03QA3ZZ, 03QA4ZZ, 03QB0ZZ, 03QB3ZZ, 03QB4ZZ,03QC0ZZ, 03QC3ZZ, 03QC4ZZ,03QY0ZZ, 03QY3ZZ,03QY4ZZ,04Q00ZZ, 04Q03ZZ, 04QC0ZZ, 04QC3ZZ  04Q04ZZ, 04QC4ZZ,04QD0ZZ, 04QD3ZZ,04QD4ZZ, 04QE0ZZ 04QE3ZZ, 04QE4ZZ, 04QF0ZZ, 04QF3ZZ, 04QF4ZZ, 04QH0ZZ, 04QH3ZZ, 04QH4ZZ,04QJ0ZZ, 04QL4ZZ, 04QY0ZZ, 04QY3ZZ  04QJ3ZZ, 04QJ4ZZ,04QK0ZZ, 04QK3ZZ, 04QL0ZZ, 04QL3ZZ, |
| Other vascular complications | [T81.719A](https://www.icd10data.com/ICD10CM/Codes/S00-T88/T80-T88/T81-/T81.719A) , [T81.72XA](https://www.icd10data.com/ICD10CM/Codes/S00-T88/T80-T88/T81-/T81.72XA)  T82.837, T82.838 |
| **Infection** |  |
| Fever | T82.6, T82.7, R50.82 |
| Septicemia | A41.9, A65.20, [T81.12XA](https://icd.codes/icd10cm/T8112XA) |
| Post-procedural pneumonia | J95.89 |
| **Neurological** |  |
| Nervous system complication, unspecified | [G97.81](https://www.icd10data.com/ICD10CM/Codes/G00-G99/G89-G99/G97-/G97.81) |
| Central nervous system complication | G97.81, G97.82 |
| Iatrogenic cerebrovascular infarction or hemorrhage | [I97.811](https://www.icd10data.com/ICD10CM/Codes/I00-I99/I95-I99/I97-/I97.811), [I97.821](https://www.icd10data.com/ICD10CM/Codes/I00-I99/I95-I99/I97-/I97.821)  I97.810 |
| Transient ischemic attack | [G45.9](https://www.icd10data.com/ICD10CM/Codes/G00-G99/G40-G47/G45-/G45.9), [I67.848](https://www.icd10data.com/ICD10CM/Codes/I00-I99/I60-I69/I67-/I67.848) |
| Any stroke | 160.9,161.9, 163.22, 163.139, 163.239  163.019,163.119, 163.219 |
| **Acute renal failure** | N17, N17.1, N17.2, N17.8, N17.9, N99.0, |
| **Cardiogenic shock** | [R57.0](https://www.icd10data.com/ICD10CM/Codes/R00-R99/R50-R69/R57-/R57.0) |
| **Diaphragmatic Paralysis** | [J98.6](https://www.icd10data.com/ICD10CM/Codes/J00-J99/J96-J99/J98-/J98.6) |
| **Re-open:** | 0W39OZZ, OW3BOZZ, OW3COZZ, OW3DOZZ, OW3QOZZ |
| **Pacemaker implantation** | OJH606Z, OJH636Z, 0JH806Z, OJH836Z, OJH60PZ, OJH63PZ, 0JH80PZ, OJH83PZ, OJH604Z, OJH634Z, OJH804Z, OJH834Z, OJH605Z, 0JH635Z, OJH805Z, 0JH835Z, 02H73KZ, O2HK3KZ,02HL3KZ,02HN0KZ, 02HN4KZ, OJH608Z, OJH638Z, OJH808Z, 0JH838Z, 02H60KZ, 02H63KZ, 02H64KZ, 02H70KZ, 02H73KZ, 02H74KZ, 02HK0KZ, 02HK3KZ, 02HK4KZ,02HL0KZ, 02HL3KZ, 02HL4KZ, 0JH608Z, 0JH638Z, 0JH808Z, 0JH838Z, 02H60KZ, 02H63KZ, 02H64KZ, 02H70KZ, 02H73KZ, 02H74KZ, 02HK0KZ, 02HK3KZ, 02HK4KZ, 02HL0KZ, 02HL3KZ, 02HL4KZ, 0JH608Z, 0JH638Z, 0JH808Z, 0JH838Z |
| **Atrial fibrillation New onset** | I97.89 **plus** I48.0 |
| **Wound Infection** | S21.3, L08.9, S21.1, S21.109A, S21.301A, S21.302A, L02.213, S21, J98.5, J85.3 |

**Appendix Table 3.** Frequency Distribution of Complications by BMI Group - CABG

| **BMI subgroups** | **< 19** | **20-25** | **26-30** | **31-35** | **36-39** | **>40** | **Total** | **P-Value** |
| --- | --- | --- | --- | --- | --- | --- | --- | --- |
| **%** |  |  |  |  |  |  |  |  |
| Acute Renal Failure | 24.1 | 18.3 | 15.3 | 15.5 | 16.5 | 20.5 | 17.0 | <.0001 |
| Atrial Fibrillation New Onset | 23.4 | 18.6 | 18.2 | 18.0 | 18.9 | 19.7 | 18.7 | 0.0001 |
| Cardiac | 12.4 | 10.3 | 9.2 | 10.1 | 10.6 | 10.3 | 10.1 | 0.017 |
| Cardiogenic Shock | 10.9 | 7.6 | 6.2 | 4.7 | 5.1 | 6.0 | 5.5 | <.0001 |
| Diaphragmatic Paralysis | 0.0 | 0.8 | 0.1 | 0.3 | 0.2 | 0.1 | 0.2 | <.0001 |
| Infection | 6.6 | 4.2 | 3.4 | 3.1 | 3.2 | 4.7 | 3.6 | <.0001 |
| Neurological | 0.7 | 0.8 | 0.4 | 0.2 | 0.2 | 0.3 | 0.3 | 0.0001 |
| New Pacemaker | 2.2 | 1.5 | 0.9 | 0.9 | 1.1 | 1.1 | 1.0 | 0.002 |
| Pericardial | 1.5 | 1.5 | 1.1 | 1.0 | 0.8 | 1.3 | 1.1 | 0.002 |
| Pulmonary | 22.6 | 11.8 | 11.5 | 9.8 | 10.3 | 12.7 | 11.1 | <.0001 |
| Re-open | 0.0 | 1.1 | 0.4 | 0.4 | 0.5 | 0.4 | 0.4 | 0.0004 |
| Vascular | 0.0 | 1.5 | 0.6 | 0.5 | 0.4 | 1.0 | 0.6 | <.0001 |
| Wound Infection | 0.0 | 0.0 | 0.0 | 0.1 | 0.1 | 0.3 | 0.1 | <.0001 |

BMI=Body Mass Index Kg/m^2^

**Appendix Table 4.** CABG Predictors of Length of Stay for Indication: CABG -Univariate

| **Predictor** | **Mean (95% CI)** | **P-Value** |
| --- | --- | --- |
| **BMI Group Kg/m^2^** |  | <.001 |
| <19 | 14.79 (13.79,15.78) | <.001 |
| 20-25 | 11.46 (10.75,12.16) | N/A |
| 26-30 | 9.18 (8.89,9.47) | <.001 |
| 31-35 | 8.79 (8.60,8.98) | <.001 |
| 36-39 | 9.00 (8.74,9.26) | <.001 |
| >40 | 10.55 (10.31,10.79) | 0.017 |
| **Age Group, years** |  | 0.580 |
| 18-44 | 8.56 (7.94,9.18) | N/A |
| 45-59 | 8.91 (8.69,9.12) | 0.296 |
| 60-74 | 9.49 (9.32,9.65) | 0.004 |
| >75 | 10.88 (10.55,11.21) | <.001 |
| **Gender** |  | <.001 |
| Male | 9.17 (9.03,9.31) | N/A |
| Female | 10.13 (9.91,10.34) | <.001 |
| **Race** |  | <.001 |
| Non-white | 10.72 (10.43,11.01) | N/A |
| White | 9.22 (9.08,9.35) | <.001 |
| **Deyo-CCI** |  | <.001 |
| 1 | 7.66 (7.40,7.92) | N/A |
| 0 | 6.72 (6.34,7.10) | <.001 |
| 2 or higher | 10.30 (10.16,10.43) | <.001 |
| ***Comorbidities*** |  |  |
| **Atrial Fibrillation/Flutter** |  | <.001 |
| No | 8.76 (8.62,8.90) | N/A |
| Yes | 10.86 (10.66,11.06) | <.001 |
| **Chronic pulmonary disease** |  | <.001 |
| No | 9.18 (9.04,9.31) | N/A |
| Yes | 10.33 (10.09,10.57) | <.001 |
| **Congestive heart failure** |  | <.001 |
| No | 9.26 (9.13,9.38) | N/A |
| Yes | 10.68 (10.36,10.99) | <.001 |
| **Diabetes Mellitus** |  | <.001 |
| No | 9.24 (9.08,9.39) | N/A |
| Yes | 9.75 (9.57,9.93) | <.001 |
| **Hypertension** |  | <.001 |
| No | 11.05 (10.85,11.25) | N/A |
| Yes | 8.65 (8.51,8.80) | <.001 |
| **Peripheral vascular disorders** |  | <.001 |
| No | 9.30 (9.17,9.42) | N/A |
| Yes | 10.41 (10.10,10.73) | <.001 |
| **Prior Myocardial Infraction** |  | 0.035 |
| No | 9.52 (9.39,9.65) | N/A |
| Yes | 9.20 (8.93,9.47) | 0.035 |
| **Chronic Renal Disease** |  | <.001 |
| No | 8.88 (8.75,9.01) | N/A |
| Yes | 11.56 (11.31,11.80) | <.001 |
| ***Clinical Course*** |  |  |
| Non-ST Elevation MI |  | <.001 |
| No | 8.63 (8.49,8.77) | N/A |
| Yes | 11.51 (11.29,11.72) | <.001 |
| ST Elevation MI |  | <.001 |
| No | 9.40 (9.28,9.52) | N/A |
| Yes | 10.55 (10.02,11.08) | <.001 |
| Percutaneous Coronary Intervention |  | <.001 |
| No | 9.39 (9.27,9.51) | N/A |
| Yes | 12.24 (11.48,13.01) | <.001 |
| Prior Sternotomy |  | 0.006 |
| No | 9.54 (9.41,9.67) | N/A |
| Yes | 9.12 (8.86,9.39) | 0.006 |
| Concomitant Surgical Valve |  | <.001 |
| No | 9.39 (9.27,9.51) | N/A |
| Yes | 11.61 (10.90,12.32) | <.001 |
| VT/VF |  | <.001 |
| No | 9.29 (9.17,9.41) | N/A |
| Yes | 12.37 (11.86,12.88) | <.001 |
|  |  |  |

BMI = Body Mass Index; Deyo-CCI = Deyo-Charlson Comorbidity Index; NSTEMI = Non-ST Segment Elevation Myocardial Infraction; STEMI = ST segment Myocardial Infraction; VF/VT = Ventricular Flutter/Ventricular Fibrillation

**Appendix Table 5.** CABG Predictors of Length of Stay for Indication: Multivariate

| **Predictor** | **Mean (95% CI)** | **P-Value** |
| --- | --- | --- |
| **BMI Group, Kg/m^2^** |  | <.001 |
| <19 | 12.95 (11.88,14.03) | <.001 |
| 20-25 | 9.94 (9.13,10.75) | N/A |
| 26-30 | 7.97 (7.52,8.43) | <.001 |
| 31-35 | 7.75 (7.36,8.14) | <.001 |
| 36-39 | 7.96 (7.53,8.39) | <.001 |
| >40 | 9.41 (8.98,9.83) | 0.180 |
| **Age Group, years** |  | <.001 |
| 18-44 | 8.30 (7.57,9.03) | N/A |
| 45-59 | 8.77 (8.34,9.20) | 0.172 |
| 60-74 | 9.48 (9.08,9.88) | <.001 |
| >75 | 10.77 (10.28,11.26) | <.001 |
| **Gender** |  | 0.002 |
| Female | 9.54 (9.10,9.99) | 0.002 |
| Male | 9.12 (8.70,9.53) | N/A |
| **Race** |  | <.001 |
| Non-white | 9.94 (9.46,10.42) | N/A |
| White | 8.72 (8.32,9.12) | <.001 |
| **Deyo-CCI** |  | <.001 |
| 0 | 8.00 (7.45,8.56) | <.001 |
| 1 | 8.85 (8.39,9.31) | N/A |
| 2 or higher | 11.14 (10.75,11.53) | <.001 |
| ***Comorbidities*** |  |  |
| Atrial Fibrillation/Flutter |  | <.001 |
| No | 8.87 (8.46,9.28) | N/A |
| Yes | 10.70 (10.25,11.15) | <.001 |
| Congestive heart failure |  | 0.006 |
| No | 9.29 (8.87,9.70) | N/A |
| Yes | 9.78 (9.26,10.30) | 0.006 |
| Chronic pulmonary disease |  | 0.081 |
| No | 9.29 (8.88,9.70) | N/A |
| Yes | 9.55 (9.07,10.02) | 0.081 |
| Diabetes Mellitus |  | 0.008 |
| No | 9.40 (8.99,9.82) | N/A |
| Yes | 9.05 (8.60,9.51) | 0.008 |
| Hypertension |  | <.001 |
| No | 10.42 (9.98,10.87) | N/A |
| Yes | 8.89 (8.47,9.30) | <.001 |
| Prior Myocardial Infraction |  | <.001 |
| No | 9.44 (9.03,9.85) | N/A |
| Yes | 8.41 (7.92,8.91) | <.001 |
| Peripheral vascular disorders |  | 0.843 |
| No | 9.33 (8.92,9.74) | N/A |
| Yes | 9.36 (8.84,9.88) | 0.843 |
| Chronic Renal Disease |  | <.001 |
| No | 9.16 (8.75,9.57) | N/A |
| Yes | 10.67 (10.18,11.16) | <.001 |
| ***Clinical Course*** |  |  |
| Non-ST Elevation MI |  | <.001 |
| No | 8.83 (8.42,9.24) | N/A |
| Yes | 11.08 (10.63,11.54) | <.001 |
| ST-Elevation MI |  | 0.010 |
| No | 9.30 (8.89,9.71) | N/A |
| Yes | 10.02 (9.36,10.69) | 0.010 |
| Percutaneous Coronary Intervention |  | <.001 |
| No | 9.31 (8.90,9.72) | N/A |
| Yes | 11.69 (10.81,12.57) | <.001 |
| Prior Sternotomy |  | <.001 |
| No | 9.42 (9.01,9.83) | N/A |
| Yes | 8.86 (8.38,9.34) | <.001 |
| Concomitant Valve surgery |  | <.001 |
| No | 9.28 (8.87,9.69) | N/A |
| Yes | 11.13 (10.32,11.95) | <.001 |
| VT/VF |  | <.001 |
| No | 9.20 (8.79,9.61) | N/A |
| Yes | 11.93 (11.28,12.57) | <.001 |

BMI = Body Mass Index; Deyo-CCI = Deyo-Charlson Comorbidity Index; NSTEMI = Non-ST Segment Elevation Myocardial Infraction; STEMI = ST segment Myocardial Infraction; VF/VT = Ventricular Flutter/Ventricular Fibrillation
